# Tumour stage distribution and survival of malignant melanoma in Germany 2002–2011

**DOI:** 10.1186/s12885-016-2963-0

**Published:** 2016-12-05

**Authors:** Olaf Schoffer, Stefanie Schülein, Gerlinde Arand, Hans Arnholdt, Dieter Baaske, Ralf C. Bargou, Nikolaus Becker, Matthias W. Beckmann, Yves Bodack, Beatrix Böhme, Tayfun Bozkurt, Regine Breitsprecher, Andre Buchali, Elke Burger, Ulrike Burger, Klaus Dommisch, Gudrun Elsner, Karin Fernschild, Ulrike Flintzer, Uwe Funke, Michael Gerken, Hubert Göbel, Norbert Grobe, Vera Gumpp, Lucie Heinzerling, Lana Raffaela Kempfer, Alexander Kiani, Monika Klinkhammer-Schalke, Sabine Klöcking, Ute Kreibich, Katrin Knabner, Peter Kuhn, Stine Lutze, Uwe Mäder, Tanja Maisel, Jan Maschke, Martin Middeke, Andreas Neubauer, Antje Niedostatek, Anabelle Opazo-Saez, Christoph Peters, Beatrice Schell, Gerhard Schenkirsch, Harald Schmalenberg, Peter Schmidt, Constanze Schneider, Birgit Schubotz, Anika Seide, Paul Strecker, Sabine Taubenheim, Matthias Wackes, Steffen Weiß, Claudia Welke, Carmen Werner, Christian Wittekind, Jörg Wulff, Heike Zettl, Stefanie J. Klug

**Affiliations:** 1 Cancer Epidemiology, University Cancer Center, Technische Universität Dresden, Dresden, Germany; 2 Epidemiology, Department of Sport and Health Sciences, Technical University of Munich, Georg-Brauchle-Ring 56, 80992 Munich, Germany; 3 Onkologischer Schwerpunkt (OSP) Göppingen, Göppingen, Germany; 4 Tumour Centre Augsburg, Augsburg, Germany; 5 Tumour Centre Chemnitz, Chemnitz, Germany; 6 Tumour Centre Würzburg, Würzburg, Germany; 7 National Centre for Tumour Diseases (NCT) Heidelberg, Heidelberg, Germany; 8 Tumour Centre Erlangen-Nürnberg, Erlangen, Germany; 9 Onkologische Qualitätssicherung (QS) in Westfalen-Lippe, Münster, Germany; 10 Regional Clinical Cancer Registry Magdeburg, Magdeburg, Germany; 11 Tumour Centre Koblenz, Koblenz, Germany; 12 Tumour Centre Vorpommern, Greifswald, Germany; 13 Tumour Centre Brandenburg, Frankfurt (Oder), Germany; 14 Tumour Centre Jena, Jena, Germany; 15 Tumour Centre Schwerin, Schwerin, Germany; 16 Suedharz Klinikum Nordhausen gGmbH, Nordhausen, Germany; 17 Tumour Centre Neubrandenburg, Neubrandenburg, Germany; 18 Tumour Centre Gera, Gera, Germany; 19 Tumour Centre Regensburg, Regensburg, Germany; 20 Tumour Centre Erfurt, Erfurt, Germany; 21 Tumour Centre Freiburg/Comprehensive Cancer Centre Freiburg (CCCF), Freiburg im Breisgau, Germany; 22 Tumour Centre Oberfranken, Bayreuth, Germany; 23 Tumour Centre Rostock, Rostock, Germany; 24 Cancer Register of Southwest Saxony, Zwickau, Zwickau, Germany; 25 Tumour Centre Ulm/Comprehensive Cancer Centre Ulm (CCCU), Ulm, Germany; 26 Tumour Centre Görlitz, Görlitz, Germany; 27 Department of Dermatology, University Hospital Carl Gustav Carus, Technische Universität Dresden, Dresden, Germany; 28 Comprehensive Cancer Centre (CCC) Marburg, Marburg, Germany; 29 Regional Clinical Cancer Registry Dresden, Dresden, Germany; 30 Klinisches Krebsregister Halle, Halle, Germany; 31 Tumour Centre Leipzig, Leipzig, Germany; 32 Tumour Centre Suhl, Suhl, Germany

**Keywords:** Malignant melanoma, TNM staging, Survival analysis, Skin cancer screening, Stage distribution

## Abstract

**Background:**

Over the past two decades, there has been a rising trend in malignant melanoma incidence worldwide. In 2008, Germany introduced a nationwide skin cancer screening program starting at age 35. The aims of this study were to analyse the distribution of malignant melanoma tumour stages over time, as well as demographic and regional differences in stage distribution and survival of melanoma patients.

**Methods:**

Pooled data from 61 895 malignant melanoma patients diagnosed between 2002 and 2011 and documented in 28 German population-based and hospital-based clinical cancer registries were analysed using descriptive methods, joinpoint regression, logistic regression and relative survival.

**Results:**

The number of annually documented cases increased by 53.2% between 2002 (*N* = 4 779) and 2011 (*N* = 7 320). There was a statistically significant continuous positive trend in the proportion of stage UICC I cases diagnosed between 2002 and 2011, compared to a negative trend for stage UICC II. No trends were found for stages UICC III and IV respectively. Age (OR 0.97, 95% CI 0.97–0.97), sex (OR 1.18, 95% CI 1.11–1.25), date of diagnosis (OR 1.05, 95% CI 1.04–1.06), ‘diagnosis during screening’ (OR 3.24, 95% CI 2.50–4.19) and place of residence (OR 1.23, 95% CI 1.16–1.30) had a statistically significant influence on the tumour stage at diagnosis. The overall 5-year relative survival for invasive cases was 83.4% (95% CI 82.8–83.9%).

**Conclusions:**

No distinct changes in the distribution of malignant melanoma tumour stages among those aged 35 and older were seen that could be directly attributed to the introduction of skin cancer screening in 2008.

**Electronic supplementary material:**

The online version of this article (doi:10.1186/s12885-016-2963-0) contains supplementary material, which is available to authorized users.

## Background

Malignant melanoma occurs primarily in fair-skinned populations, with almost 80% of new cases worldwide occurring in North America, Europe, Australia and New Zealand [1]. It is the fourth most common cancer in Australia (age-standardised incidence rate 34.9 per 100 000, World Standard Population), the sixth most common cancer in North America (13.8 per 100 000) and the seventh most common cancer in the European Union (10.2 per 100 000), as well as in Germany (11.4 per 100 000) [2]. Although the age-standardised incidence rate in Germany increased considerably more between 2006 and 2008 than in other years since 1999, malignant melanoma-related mortality has remained relatively stable over the same period [3].

Malignant melanoma can be treated with a very good prognosis if it is detected in the early stages (Union internationale contre le cancer (UICC) 0-I). However, in advanced stages, therapies which were available up until 2011 had limited effect.

A screening program for skin cancer was set up in the USA in 1985, with national screening and educational programs having expanded to all 50 states [4]. Although examinations for early detection of skin cancer were already offered in Germany since 1971 [5], Germany was the first country in Europe to introduce nationwide skin cancer screening in 2008 [6]. Since 1st July 2008, all men and women aged 35 years and older have been eligible for a skin examination by a dermatologist or specially certified physician every two years. However, the screening is not organised and there is no invitation to attend. According to data reported for Germany, participation among those aged 35 years and older was around 30% between 2008 and 2010 [7, 8].

A regional German pilot project in the northern federal state of Schleswig-Holstein conducted between July 2003 and June 2004 provided preliminary evidence for the effectiveness of screening [9]. Changes in TNM stage-specific incidence were shown, with an increase in prognostically favourable malignant melanoma, including in situ and pT1. The incidence of advanced malignant melanoma, including pT2, pT3 and, for women only, pT4 substantially declined [9, 10].

We analysed data from 61 895 malignant melanoma patients diagnosed between 2002 and 2011 and documented in German clinical cancer registries. This period was prior to the introduction of targeted therapies. Clinical characteristics of malignant melanoma were investigated and proportional changes, as well as demographic and regional differences in the distribution of tumour stages were described over time. We examined the survival of melanoma patients, overall and stratified by age, sex, UICC stage, ‘diagnosis during screening’ and place of residence.

## Methods

A population-based clinical registry structure in eastern Germany and Bavaria has been in existence for about two decades, while in the other German federal states these registries are predominantly still in the development stage. Population-based clinical registries, as opposed to hospital-based clinical registries, have a regional focus, registering cases resident in their respective catchment areas. Particularly in the five eastern German federal states and Bavaria, it is mandatory that data of cases registered at the clinical cancer registries are then passed on to the respective epidemiological state registry. The epidemiological cancer registries in Germany have nationwide coverage.

In June 2013, all clinical cancer registries in Germany were contacted by the Working Group of German Tumour Centres and Clinical Cancer Registries (ADT) and were asked to provide anonymised data on malignant melanoma patients diagnosed between 2002 and 2011. Data from 24 population-based registries and four hospital-based registries from Baden-Württemberg, Bavaria, Brandenburg, Hesse, Mecklenburg-Western Pomerania, North Rhine-Westphalia, Rhineland-Palatinate, Saxony, Saxony-Anhalt and Thuringia were included in the analyses. Regarding hospital-based clinical cancer registries, the place of residence of patients did not necessarily correspond to the federal state of the registry where the case was registered. Our analyses therefore include data on malignant melanoma cases resident in all federal states in Germany.

For each of the federal states Bavaria, Brandenburg, Mecklenburg-Western Pomerania, Saxony, Saxony-Anhalt and Thuringia, the number of invasive cases included in this analysis represented approximately 85% of all the newly diagnosed cases reported by the association of population-based epidemiological cancer registries (GEKID) during each year between 2002 and 2011 [11]. For all other federal states and the region Westphalia-Lippe, the proportion was less than 10%, respectively. Of the 50 446 patients with invasive tumours included in these analyses, 41 102 (81%) lived in the six federal states of Bavaria, Brandenburg, Mecklenburg-Western Pomerania, Saxony, Saxony-Anhalt and Thuringia.

Information was available on age, sex, place of residence, vital status, date of diagnosis, ‘diagnosis during screening’ (i.e. whether or not the diagnosis was made during screening) and TNM characteristics. UICC stages were derived from TNM according to the 6th edition of TNM Classification of Malignant Tumours [10], including stage UICC 0 (in situ), I, II (both invasive without metastases), III (invasive with regional lymph node metastases) and IV (invasive with distant metastases). Cases where the UICC stage could not be determined were classified as UICC X. pT stages are classified according to the following tumour thicknesses: pT1 (≤1 mm), pT2 (1.01–2.0 mm), pT3 (2.01–4.0 mm) and pT4 (>4 mm). It should be noted that there was a change in the TNM classification between 2001 and 2002, with the tumour thickness of 1.5–2.0 mm being reclassified from stage UICC II to stage UICC I.

### Inclusion and exclusion criteria

Both population-based (*n* = 24) and hospital-based clinical cancer registries (*n* = 7) provided data. All malignant melanoma cases that were diagnosed between 2002 and 2011, with localisation of ICD-O C44.0-C44.9 (ICD-10: C43.0-C43.9 and D03.0-D03.9) and histology of ICD-O M8720-M8790, were included [12]. Only cases aged 15 years and above at diagnosis were included in the analysis, as younger cases were registered by a separate childhood cancer registry. Additionally, only primary melanoma diagnoses were considered. Three registries that did not document data for the entire period of observation (2002–2011, regarding date of diagnosis) were excluded from the analyses. Cases were also excluded when the date of diagnosis documented in the registry was identical to the date of death retrieved from a death certificate (death certificate only, DCO) and when the sex of the patient was not known (Additional file 1: Figure S1).

### Statistical methods

Descriptive analyses as well as logistic regressions and survival estimations were performed. Patients were categorised into the age groups 15–34 years, 35–49 years, 50–64 years, 65–79 years and over 79 years. Additionally, to emphasise the difference between younger and older patients, the age groups were further aggregated into the categories 15–34 years, 35–64 years and 65 years and older.

Absolute numbers and proportions of UICC tumour stages were described over time (2002–2011).

#### Joinpoint regression

Tests for trends in age-specific proportions of UICC stages over time were performed using joinpoint regression. This method describes changing trends over successive segments of time, and the extent of increase or decrease within each segment [13]. We investigated whether the identified trends were continuous for the complete time period (2002–2011) or whether changing trends for specific segments could be identified. APC (annual percent change) and the respective 95% confidence intervals were estimated to indicate the magnitude of the trends.

#### Logistic regression

Univariable and multivariable binary logistic regression [14] was used to model the associations between the chance of being diagnosed at stage UICC I vs. UICC II–IV and potential influencing factors, including age, sex, date of diagnosis, ‘diagnosis during screening’ and geographical area of residence (eastern Germany including Berlin, Brandenburg, Mecklenburg-Western Pomerania, Saxony, Saxony-Anhalt and Thuringia vs. western Germany including Baden-Württemberg, Bavaria, Bremen, Hamburg, Hesse, Lower Saxony, North Rhine-Westphalia, Rhineland-Palatinate, Saarland and Schleswig Holstein). In the logistic regression, cases with UICC 0 or UICC X and cases from registries that did not collect any data for the variable ‘diagnosis during screening’ (registries with 100% missing values for the variable ‘diagnosis during screening’) were excluded. Since only men and women aged 35 years and older are eligible for skin cancer screening, cases diagnosed at younger ages were also excluded from the logistic regression models.

Variable selection was performed such that only variables with significant influence in the univariable models were included in the multivariable model. The variable selection was verified by Wald test and bootstrapping [15]. The bootstrap procedure was performed with 1 000 runs and 100*(1-α)% confidence intervals were estimated from the α/2 and (1-α/2) percentile of the 1 000 resulting parameter estimators.

In order to assess the impact of missing values for the variable ‘diagnosis during screening’, the multivariable logistic regression models were also performed as complete case analysis (i.e. exclusion of all cases without information about whether the diagnosis was made during screening). Additionally, to include all available patient data, a multiple imputation [16] for the variable ‘diagnosis during screening’ was performed using a discriminant function by fully conditional specification method [17]. A logistic regression model was also conducted without the ‘diagnosis during screening’ variable.

#### Survival estimation

Only population-based registries were included in the survival analysis in order to ensure consistency with regard to how information on follow-up was captured and to perform a population-based analysis. Survival estimations were carried out based on the Nelson-Aalen estimator [18] with the cohort approach. Relative survival rates were derived using the Ederer II method [19, 20]. The median survival was estimated as the lowest survival time where the overall survival function was ≤ 0.5. Stratified survival for patients with invasive tumours (UICC 0 excluded) was estimated according to age group (15–34, 35–49, 50–64, 65–79 and over 79 years), sex, UICC stage, ‘diagnosis during screening’ and place of residence. Differences between the relative survival for different strata were tested by pairwise Log-rank test [21].The following reference categories were chosen - age: 65–79 years, sex: female, UICC stage: I, ‘diagnosis during screening’: no, place of residence: eastern Germany. Confidence intervals for specific survival rates were estimated by log-log transformation [22] of specific standard errors according to Greenwood's formula [23]. Even though the Nelson-Aalen estimator already takes censored values into account, it is possible that differences in vital status can bias survival estimates. Multiple imputation of vital status for cases lost to follow-up, using a cox regression model, was therefore applied according to Carpenter and Kenward [24].

All test results were considered statistically significant if *p* < 0.05. Descriptive analyses, logistic regression and relative survival were performed with SAS statistical analysis software (Version 9.3, Cary, North Carolina, USA). Joinpoint regression was performed with the Joinpoint Regression Program (Version 4.2.0.2, Statistical Research and Applications Branch, National Cancer Institute, Bethesda, Maryland, USA).

## Results

### Study Population

**Fig. 1:**
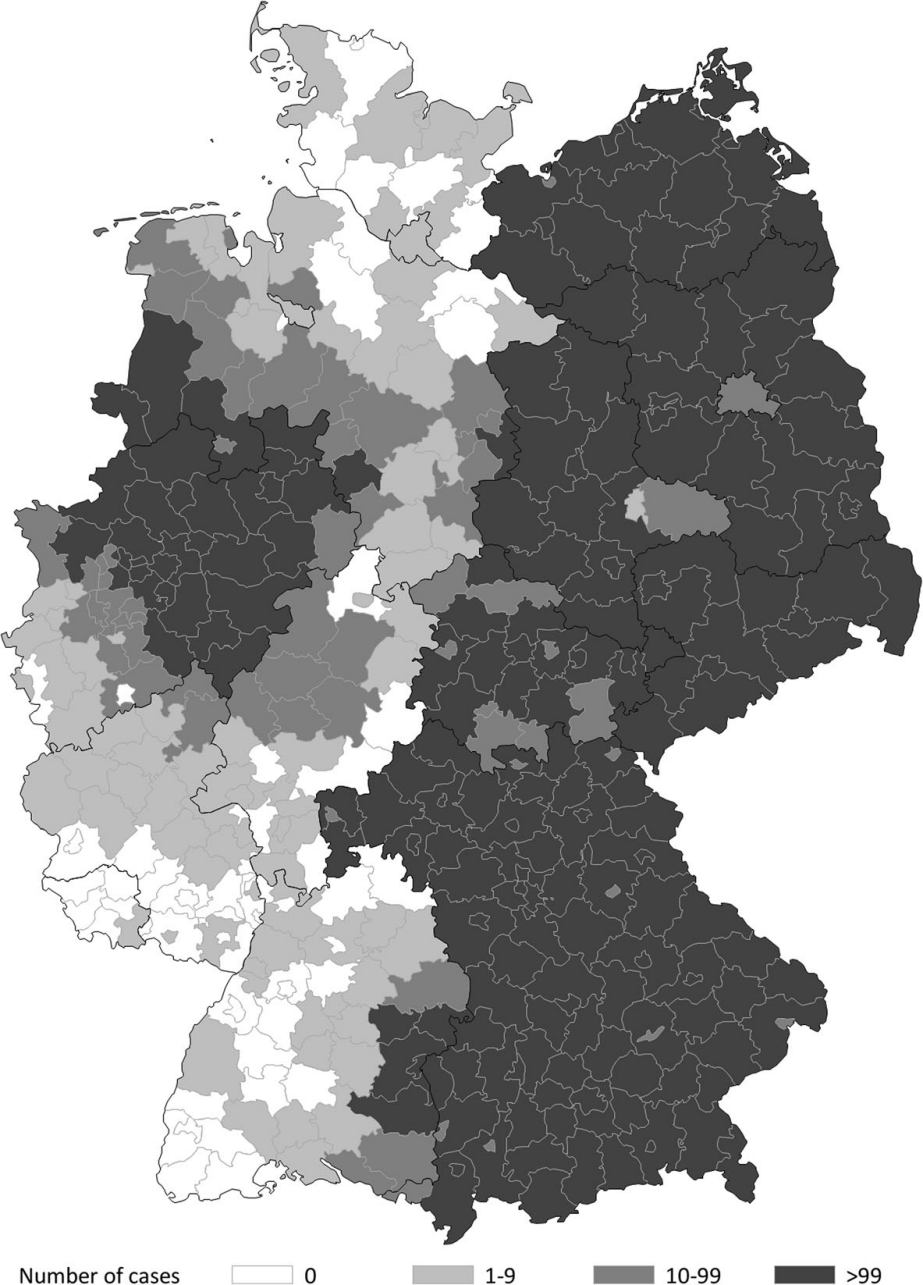
Regional distribution of malignant melanoma cases by place of residence between 2002 and 2011 in Germany, *N* = 61 895

A total of 61 895 cases with a primary malignant melanoma registered in 24 population-based and four hospital-based clinical cancer registries in Baden-Württemberg, Bavaria, Brandenburg, Hesse, Mecklenburg-Western Pomerania, North Rhine-Westphalia, Rhineland-Palatinate, Saxony, Saxony-Anhalt and Thuringia were included in the analyses (Fig. 1).

**Fig. 2:**
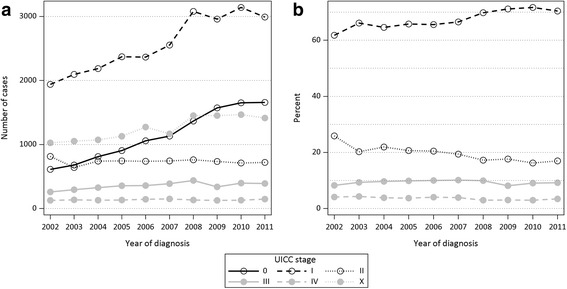
Distribution of UICC stages between 2002 and 2011. **a** Number of cases, stages UICC 0-IV and X, *N* = 61 895. **b** Proportions of malignant melanoma patients, stages UICC I-IV, *N* = 37 947

The number of annually documented melanoma cases (including UICC Stage 0 and X) increased by 53.2% over time, from 4 779 in 2002 to 7 320 in 2011. The largest increase in documented cases occurred between 2007 (6 134 cases) and 2008 (7 229 cases) (Table 1). The number of cases diagnosed with stage UICC 0 increased continuously over time. Stage UICC I also increased over time, with the largest increase in number of cases occurring between 2007 and 2008. Stage UICC II showed a decline in number of cases (2002: 814 cases; 2011: 720 cases), although from 2003 onwards the number of cases remained relatively constant. Stage UICC III showed an increase in number of cases (2002: 260 cases; 2011: 392 cases) while stage UICC IV only showed a slight increase over time (2002: 128 cases; 2011: 147 cases) (Table 1, Fig. 2a).

**Table 1:** Malignant melanoma patients diagnosed between 2002 and 2011 by age at diagnosis, sex, year of diagnosis and UICC stage, *N* = 61 895

		Stage						Total
Stratum		UICC 0	UICC I	UICC II	UICC III	UICC IV	UICC X	N (%)^b^
		N (%) {%}^a^						
Total		11 449 (18.5)	25 681 (41.5)	7 352 (11.9)	3 555 (5.7)	1 359 (2.2)	12 499 (20.2)	61 895 (100.0)
			{67.7}	{19.4}	{9.4}	{3.6}		
Age at diagnosis (years)	15–34	764 (15.5)	2 562 (52.1)	346 (7.0)	252 (5.1)	48 (1.0)	942 (19.2)	4 914 (7.9)
			{79.9}	{10.8}	{7.9}	{1.5}		
	35–49	1 811 (15.2)	5 864 (49.3)	965 (8.1)	712 (6.0)	216 (1.8)	2 320 (19.5)	11 888 (19.2)
			{75.6}	{12.4}	{9.2}	{2.8}		
	50–64	2 913 (17.4)	7 385 (44.1)	1 713 (10.2)	1 036 (6.2)	401 (2.4)	3 296 (19.7)	16 744 (27.1)
			{70.1}	{16.3}	{9.8}	{3.8}		
	65–79	4 704 (21.2)	8 167 (36.9)	3 092 (14.0)	1 243 (5.6)	521 (2.4)	4 419 (20.0)	22 146 (35.8)
			{62.7}	{23.7}	{9.5}	{4.0}		
	≥80	1 257 (20.3)	1 703 (27.5)	1 236 (19.9)	312 (5.0)	173 (2.8)	1 522 (24.5)	6 203 (10.0)
			{49.7}	{36.1}	{9.1}	{5.1}		
Sex	Male	5 165 (17.0)	12 542 (41.2)	3 807 (12.5)	2 065 (6.8)	823 (2.7)	6 065 (19.9)	30 467 (49.2)
			{65.2}	{19.8}	{10.7}	{4.3}		
	Female	6 284 (20.0)	13 139 (41.8)	3 545 (11.3)	1 490 (4.7)	536 (1.7)	6 434 (20.5)	31 428 (50.8)
			{70.2}	{18.9}	{8.0}	{2.9}		
Year of diagnosis	2002	612 (12.8)	1 939 (40.6)	814 (17.0)	260 (5.4)	128 (2.7)	1 026 (21.5)	4 779 (7.7)
			{61.7}	{25.9}	{8.3}	{4.1}		
	2003	674 (14.0)	2 095 (42.8)	642 (13.1)	296 (6.0)	136 (2.8)	1 051 (21.4)	4 900 (7.9)
			{66.1}	{20.3}	{9.3}	{4.3}		
	2004	805 (15.5)	2 187 (41.5)	742 (14.1)	327 (6.2)	130 (2.5)	1 074 (20.4)	5 271 (8.5)
			{64.6}	{21.9}	{9.7}	{3.8}		
	2005	897 (16.1)	2 371 (42.1)	745 (13.2)	355 (6.3)	133 (2.4)	1 129 (20.0)	5 638 (9.1)
			{65.8}	{20.7}	{9.9}	{3.7}		
	2006	1 052 (18.0)	2 366 (39.8)	738 (12.4)	360 (6.1)	145 (2.4)	1 272 (21.4)	5 940 (9.6)
			{65.6}	{20.4}	{10.0}	{4.0}		
	2007	1 121 (18.7)	2 553 (41.6)	744 (12.1)	390 (6.4)	150 (2.4)	1 166 (19.0)	6 134 (9.9)
			{66.5}	{19.4}	{10.2}	{3.9}		
	2008	1 357 (19.2)	3 079 (42.6)	761 (10.5)	438 (6.1)	132 (1.8)	1 451 (20.1)	7 229 (11.7)
			{69.8}	{17.3}	{9.9}	{3.0}		
	2009	1 550 (22.1)	2 960 (41.2)	734 (10.2)	339 (4.7)	127 (1.8)	1 451 (20.2)	7 184 (11.6)
			{71.2}	{17.6}	{8.1}	{3.1}		
	2010	1 631 (22.3)	3 141 (41.9)	712 (9.5)	398 (5.3)	131 (1.7)	1 466 (19.5)	7 500 (12.1)
			{71.7}	{16.2}	{9.1}	{3.0}		
	2011	1 625 (22.9)	2 990 (40.8)	720 (9.8)	392 (5.4)	147 (2.0)	1 413 (19.3)	7 320 (11.8)
			{70.4}	{16.9}	{9.2}	{3.5}		
Diagnosis during Screening	Yes	438 (36.9)	576 (48.5)	51 (4.3)	16 (1.3)	2 (0.2)	105 (8.8)	1 188 (1.9)
			{89.3}	{7.9}	{2.5}	{0.3}		
	No	3 639 (20.3)	7 832 (43.7)	2 579 (14.4)	963 (5.4)	377 (2.1)	2 532 (14.1)	17 922 (29.0)
			{66.6}	{21.9}	{8.2}	{3.2}		
	Unknown	7 372 (17.2)	17 273 (40.4)	4 722 (11.0)	2 576 (6.0)	980 (2.3)	9 862 (23.1)	42 785 (69.1)
			{67.6}	{18.5}	{10.1}	{3.8}		
Place of residence	Eastern Germany	5 531 (22.8)	10 167 (41.9)	3 196 (13.2)	1 251 (5.2)	435 (1.8)	3 672 (15.1)	24 252 (39.2)
			{67.6}	{21.2}	{8.3}	{2.9}		
	Western Germany	5 918 (15.7)	15 514 (41.2)	4 156 (11.0)	2 304 (6.1)	924 (2.5)	8 827 (23.4)	37 643 (60.8)
			{67.8}	{18.2}	{10.1}	{4.0}		

^a^percentages refer to row

{} refers to proportions of stages I–IV

^b^percentages refer to column

The largest number of patients were diagnosed between the ages of 65–79 years (*n* = 22 146, 35.8%) and the lowest number among those aged 15–34 (*n* = 4 914, 7.9%) (Table 1). A total of 30 467 men (49.2%) were diagnosed with malignant melanoma, compared to 31 428 women (50.8%). In the 50 to 79 years age group, there were more cases among men (*n* = 21 316, 54.8%) than women (*n* = 17 574, 45.2%). A distinct proportional change towards older age at diagnosis was observed over time; the proportion of new cases in the age group 65 years and older increased from 1 765 in 2002 to 3 564 in 2011 (data not shown).

### Tumour stage distribution

The proportion of cases diagnosed with stage UICC 0 increased from 12.8% in 2002 to 22.9% in 2011 (Table 1). During the period 2002–2011, the proportions of cases diagnosed with pT1, pT2, pT3 and pT4 were 40.0, 13.3, 10.3 and 7.3% respectively (Additional file 2: Table S1). The proportion of stage UICC X remained approximately 20% between 2002 and 2011 (Table 1).

For the analysis of proportional changes in stage distribution, only those cases diagnosed with stage UICC I to IV were included. The proportion of cases with UICC I increased from 61.7% in 2002 to 70.4% in 2011 (APC = 0.95, 95% CI 0.52–1.38), while the proportion of stage UICC II decreased from 25.9% in 2002 to 16.9% in 2011 (APC = −4.33, 95% CI −5.78 to −2.87) (Table 1, Fig. 2b). No statistically significant trends were found for stage UICC III and IV between 2002 and 2011.

**Fig. 3:**
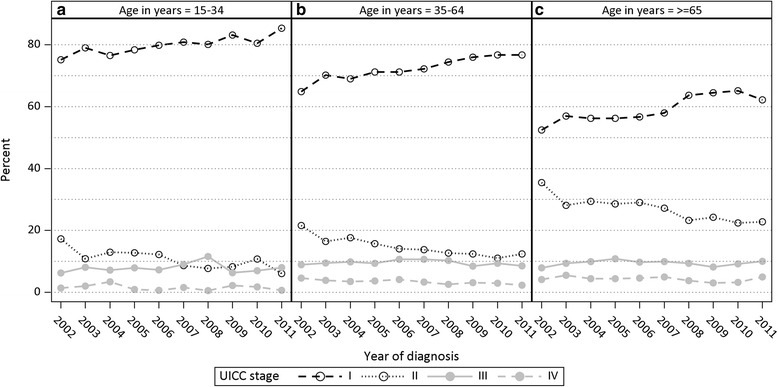
Proportion of malignant melanoma cases with stages UICC I to IV (stages UICC 0 and X excluded), by year of diagnosis and age (**a** 15–34 years, **b** 35–64 years, **c** 65 years and older), *N* = 37 947

There was a distinct increase in proportion of cases diagnosed with stage UICC I between 2008 and 2009 in the age group 15 to 34 years. The proportion of cases with stage UICC I increased continuously from 64.9% in 2002 to 76.7% in 2011 for the age group 35–64 years at diagnosis, while for those aged 65 years and older the proportion increased from 52.4 to 62.2% (Fig. 3). In the age group 65 years and older, there was a distinct increase in the proportion of cases diagnosed with stage UICC I between 2007 (57.9%) and 2008 (63.7%).

Joinpoint regression showed a significant positive trend for the complete time period (2002–2011) for the proportion of stage UICC I for all age groups: 15–34 years (APC = 1.10, 95% CI 0.62–1.58); 35–64 years (APC = 1.63, 95% CI 1.21–2.05); 65+ years (APC = 2.18, 95% CI 1.16–3.20). A significant negative trend was found for the complete time period for the proportion of stage UICC II for all age groups: 15–34 years (APC = −7.78, 95% CI −11.96 to −3.39); 35–64 years (APC = −6.31, 95% CI −8.18 to −4.40); 65+ years (APC = −4.38, 95% CI −5.87 to −2.87). No significant trends were found for UICC stage III and IV for any of the age groups, except for UICC IV among the 35 to 64 year-olds (APC = −5.52, 95% CI −8.20 to −2.76).

Joinpoint regression only revealed significant continuous trends over the entire period 2002–2011 in the different age groups, but no significant trends were revealed for distinct segments within the 2002–2011 period.

The proportion of cases diagnosed with stage UICC II over the years was substantially higher among those 65 years and older than among the younger age groups, while the proportion of cases diagnosed with stage UICC I decreased with increasing age (Fig. 3). The proportion of stage UICC II diagnosed between 2002 and 2011 increased with age from 10.8% in the age group 15–34 years to 26.3% in the age group 65 years and older, while the proportion of UICC I decreased from 79.9 to 60.0% in these two age groups respectively (Fig. 3).

Among women, the overall proportion of stage UICC I diagnosed was 70.2%, while the proportion of stage UICC II was 18.9%. Among men, these proportions were 65.2 and 19.8% respectively. The overall proportions of stage UICC III were 10.7% among men and 8.0% among women, while the overall proportions of stage UICC IV were 4.3% among men and 2.9% among women (Table 1).

The proportions of stages UICC I, II, III and IV diagnosed in eastern Germany were 67.6, 21.2, 8.3 and 2.9% respectively, compared to 67.8, 18.2, 10.1 and 4.0% in western Germany.

### Factors influencing stage at diagnosis

To investigate the effect of age, sex, date of diagnosis, diagnosis during participation in skin cancer screening and place of residence on the chance of being diagnosed with malignant melanoma at the prognostically favourable stage UICC I compared to less favourable stages UICCC II to IV, univariable and multivariable logistic regression models were conducted.

In the univariable logistic regression, all explanatory variables investigated (age, sex, date of diagnosis, ‘diagnosis during screening’ and place of residence) had a statistically significant influence (*p* < 0.0001) on the tumour stage at diagnosis (Table 2). Hence, all variables were included in the multivariable logistic regression model.

**Table 2:** Chance of being diagnosed at stage I compared to stages II to IV; for malignant melanoma patients aged 35 years and above, *N* = 23 424 (UICC 0 and X excluded; registers with 100% missing values for ‘diagnosis during screening’ excluded)

		Univariable logistic regression			Multivariable logistic regression^c^		
Parameter		Odds ratio	95% CI^a^	*p* value^b^	Odds ratio	95% CI^a^	*p* value^b^
Age at diagnosis (years)		0.97	[0.97; 0.97]	<.0001	0.97	[0.97; 0.97]	<.0001
Sex	Male	reference			reference		
	Female	1.20	[1.14; 1.27]	<.0001	1.18	[1.11; 1.25]	<.0001
Date of diagnosis (year)		1.04	[1.03; 1.05]	<.0001	1.05	[1.04; 1.06]	<.0001
Diagnosis during screening	No/unknown	reference			reference		
	Yes	3.65	[2.83; 4.71]	<.0001	3.24	[2.50; 4.19]	<.0001
Place of residence	Eastern Germany	reference			reference		
	Western Germany	1.28	[1.21; 1.35]	<.0001	1.23	[1.16; 1.30]	<.0001

^a^ *CI*: Confidence interval

^b^Wald test

^c^Multivariable model includes all listed variables

In the multivariable logistic model (Table 2), the chance of diagnosis at a prognostically favourable stage (UICC I) decreased with increasing age (OR = 0.97, 95% CI 0.97–0.97). Women were more likely to have a favourable prognosis than men (OR = 1.18, 95% CI 1.11–1.25). Diagnosis with a favourable stage was more likely in the recent calendar years (OR = 1.05, 95% CI 1.04–1.06). The likelihood of diagnosis with a favourable stage was also greater if the diagnosis was made during screening (OR = 3.24, 95% CI 2.50–4.19). Compared to eastern Germany, patients in western Germany were more likely to be diagnosed with a prognostically favourable stage (OR = 1.23, 95% CI 1.16–1.30).

The bootstrap percentile based confidence intervals confirmed the results of the multivariable logistic regression. The proportion of missing values for the variable ‘diagnosis during screening’ was however quite high, with over two thirds (69.1%) having no information. Differences were found in the structure of the missing values of the ‘diagnosis during screening’ variable in relation to place of residence between eastern and western Germany (Additional file 3: Table S2). Complete case analysis as well as multiple imputation of missing values for the variable ‘diagnosis during screening’ were performed, with the odds ratio decreasing with multiple imputation. Based on complete case analysis, the adjusted odds ratio for ‘diagnosis during screening’ was 3.57 (95% CI 2.75–4.64) and for date of diagnosis 1.04 (95% CI 1.03–1.05). Using multiple imputation for the variable ‘diagnosis during screening’, the odds ratio was 1.73 (95% CI 1.51–1.98) and for date of diagnosis 1.04 (95% CI 1.03–1.05) (data not shown). When the variable ‘diagnosis during screening’ was removed from the multivariable analysis, the odds ratio for the remaining variables also did not change considerably (age: 0.97, 95% CI 0.97–0.97; sex: 1.17, 95% CI 1.11–1.24; date of diagnosis: 1.05, 95% CI 1.04–1.06; place of residence: 1.23, 95% CI 1.16–1.30).

### Relative survival

**Fig. 4:**
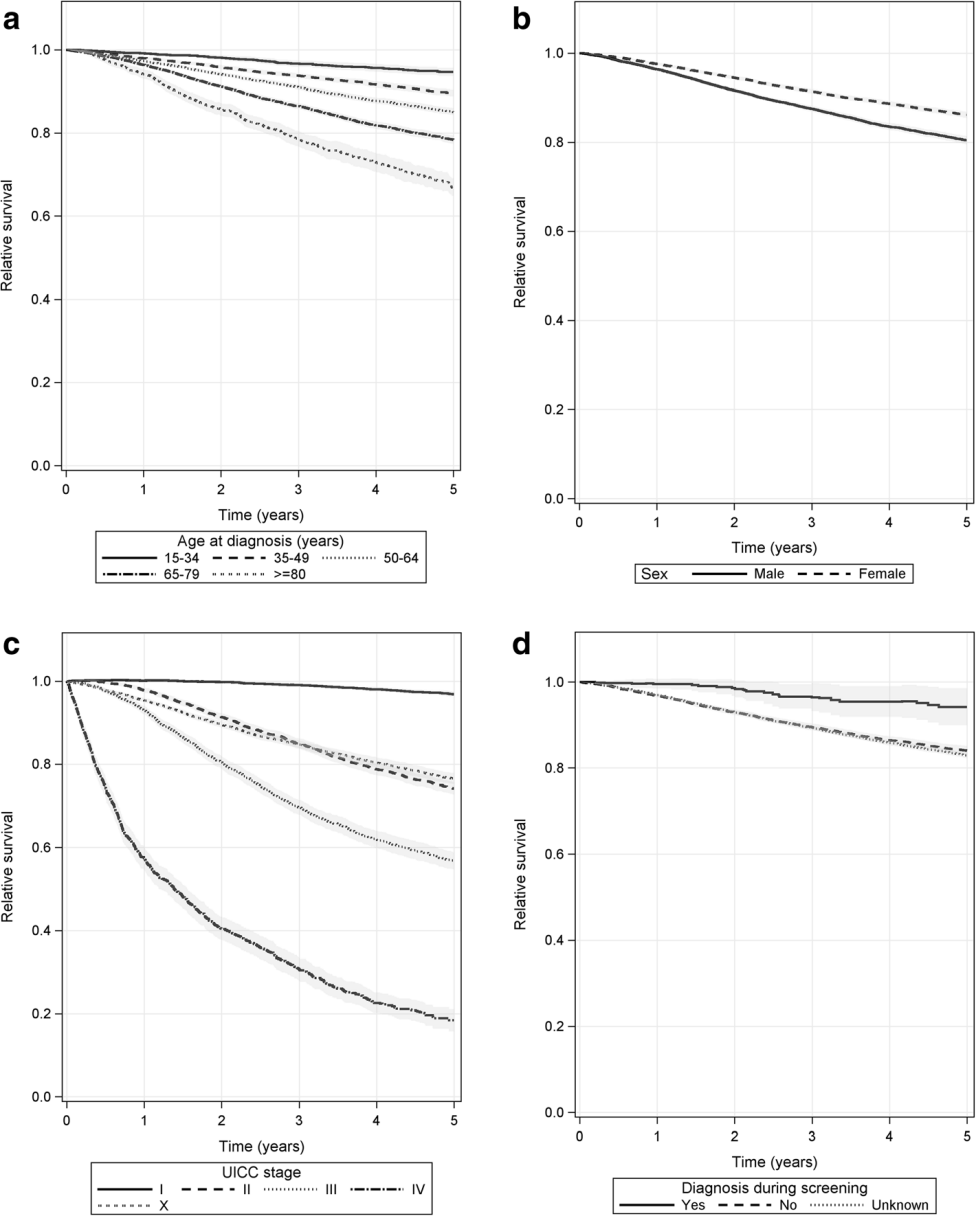
Relative 5-year survival of invasive malignant melanoma patients (UICC 0 excluded) diagnosed between 2002 and 2011, stratified by age (**a**), sex (**b**), UICC stage (**c**) and ‘diagnosis during screening’ (**d**), *N* = 49 351

Among patients with invasive tumours, women had a higher relative 5-year survival rate (86.2%, 95% CI 85.5–86.8%) than men (80.5%, 95% CI 79.6–81.4%) (Fig. 4, Additional file 4: Table S3). The relative 5-year survival rate decreased with age at diagnosis from 94.6% (95% CI 93.7–95.6%) at 15–34 years to 66.9% (95% CI 64.8–69.1%) at age 80 years and older (Fig. 4, Additional file 4: Table S3). The 5-year survival of cases with stage UICC I (96.8%, 95% CI 96.2–97.5%) was over 13 percentage points higher than the survival of all patients with invasive tumours (83.4%, 95% CI 82.8–83.9%), while relative survival of the stages UICC II, III and IV was 74.2% (95% CI 72.8–75.6%), 56.7% (95% CI 54.6–58.8%) and 18.4% (95% CI 15.8–21.1%), respectively (Fig. 4, Additional file 4: Table S3).

The relative 5-year survival rate after ‘diagnosis during screening’ was 94.2% (95% CI 89.9–98.5%) which was higher than for other cases (diagnosis not during screening: 84.1%, 95% CI 83.0–85.1%; unknown: 82.9%, 95% CI 82.3–83.6%) (Fig. 4, Additional file 4: Table S3). No differences in survival were found regarding place of residence. In eastern Germany, the relative 5-year survival rate was (83.9%, 95% CI 83.0–84.8%) and in western Germany (83.0%, 95% CI 82.4–83.7%) (Additional file 4: Table S3).

## Discussion

An increasing burden of melanoma of the skin has also been reported for Europe as well as worldwide over the past decades [25]. This pooled analysis of clinical registry data showed that the total number of malignant melanoma cases diagnosed increased in Germany between 2002 and 2011. A continuous increase in incidence over the same period has been reported based on data of the epidemiological cancer registries, which, for eastern Germany and Bavaria are to a large extent based on the same cases as were used in our analyses [3].

In addition, cancer documentation and registration, particularly of stage UICC 0, which has traditionally not been well documented, has improved in recent years. Thus, the increase in the absolute number of cases with melanoma in situ diagnoses may be due to this improved documentation. The proportion of cases diagnosed with stage UICC X remained consistently high between 2002 and 2011. Efforts therefore need to be directed towards improving the quality of documentation among physicians.

Although early detection examinations for skin cancer have been taking place in Germany since 1971, a nationwide skin cancer screening program was first introduced in July 2008 [5]. Uptake of screening in Germany has however not been high, with only around 30% of eligible individuals having participated in skin cancer screening between July 2008 and July 2010 [8]. The German population is characterised by an increasing proportion of older individuals, who are typically diagnosed at more advanced UICC stages. The effect of demographic change in terms of potentially diminishing the effectiveness of skin cancer screening in Germany should therefore also be taken into consideration.

Logistic regression models show that the variables ‘diagnosis during screening’ and the date of diagnosis had a significant impact on the stage at diagnosis. Tumours that were diagnosed during screening (OR = 3.24, 95% CI 2.50–4.19) as well as in the recent calendar years (OR = 1.05, 95% CI 1.04–1.06) were more likely to be detected in stage UICC I. It can therefore be concluded that there was a continuous proportional change in terms of stage at diagnosis over time, rather than a proportional change which could solely be attributed to the introduction of skin cancer screening in 2008.

Data for other industrialized countries showed similar stage distributions over the past decades. In a study conducted in the United States, 70% of malignant melanoma cases diagnosed between 1988 and 2006 had a thickness of pT1, compared to 5% with a thickness of pT4 [26]. In an Australian study, 40% of cases diagnosed between 1990 and 2006 were classified as pT1, compared to 3% classified as pT4 [27]. Similarly, in our study, 40% of cases diagnosed between 2002 and 2011 were classified as pT1, while 7% were classified as pT4.

Our pooled analysis showed that between 2002 and 2011, there was a statistically significant positive trend in the proportion of cases diagnosed at stage UICC I and a statistically significant negative trend in the proportion of cases with UICC II. No statistically significant trends were found for stages UICC III and IV between 2002 and 2011.

Joinpoint regression showed that there was a significant continuous positive trend for the proportion of stage UICC I and a significant continuous negative trend for the proportion of stage UICC II in all age groups for the entire period 2002–2011. No significant trends were found for any of the stages with regards to distinct time period segments. Joinpoint regression therefore shows that among those aged 35–64 years, an age group which should derive a benefit from screening, the proportion of stage UICC I rose steadily, with no distinct increase following the introduction of screening in 2008. The lack of significant trends, however, does not necessarily mean that screening had no effect on detecting earlier stages. We confirmed considerable gender differences in the distribution of age at diagnosis. Possible explanations include that melanoma is more likely to occur at an older age among men than women [28–30], or that the same tumours were diagnosed later among men than among women. This may be due to the fact that with regard to general health seeking behaviour, men are less likely to attend general health checks than women [31]. The older age at diagnosis may explain the less favourable prognoses concerning the UICC stages among men.

The pooled analysis showed that relative survival was dependent on age, sex, tumour stage and ‘diagnosis during screening’. The differences which were found with regard to overall relative 5-year survival are in line with other data for Germany [32, 33]. The overall relative 5-year survival rate in our study was 85.8%, compared to 87.3% in an analysis based on data from the Saarland Cancer Registry (*n* = 14 192) [32] and 89.4% based on data from cancer registries covering 12 out of 16 federal states and the Münster administrative district of North Rhine-Westphalia (*n* = 37 129) [33]. Our pooled analysis, however, was based on a larger data set and provides more extensive analyses with regard to a wide range of variables. In countries such as the USA, Australia, UK and the Nordic countries, findings were also similar with regard to relative survival depending on stage [34–38]. The 5-year survival rates are found to be higher among women than men. This has been shown in other studies conducted in Germany, the United States and the Netherlands [39–42].

The survival rate for those cases diagnosed during screening was higher than for the other cases. Selection bias may however have been introduced as there was a high number of missing values. Lead time bias also needs to be taken into consideration, with our data not being able to provide conclusive evidence in terms of screening having a positive effect on survival.

Significant differences in survival were not found between western and eastern Germany. There are however variations in the extent of follow-up between the registries, differences in age structure and genetic background of the populations and differences in incidence rates between these two regions [11]. It should however be noted that data from western Germany included in the analysis were predominantly from Bavaria. In another German study based on data provided by population-based epidemiological cancer registries in both eastern and western Germany for the time period 2002–2006, the 5-year relative survival for malignant melanoma was 3.3 percentage points lower in eastern compared to western Germany [43]. Our analysis did not reveal any significant differences between eastern and western Germany for this time period (2002–2006).

### Strengths and limitations

The analysed data originated from population-based and hospital-based clinical cancer registries in Germany, providing an extensive database for malignant melanoma. Clinical cancer registry data have the advantage of providing information on various clinical parameters which epidemiological cancer registries are not able to provide. For the first time in Germany, analyses on malignant melanoma were based on such a large data set and proportional changes in tumour stages were investigated over time. However, not all regions in Germany were included in this analysis due to some federal states not having data from clinical cancer registries for this time period and some registries not providing their data.

The results of our analyses are robust with regard to complete case analysis. However, multiple imputation substantially changed the OR for ‘diagnosis during screening’, most likely due to the high number of missing values for this variable. Nevertheless, the association remained statistically significant. Results relating to the ‘diagnosis during screening’ variable should be treated with caution.

Further research and evaluation regarding the effects of skin cancer screening in Germany is needed. In addition, improvement in data quality in terms of more complete notifications and documentation, particularly with regard to capturing data on whether or not the diagnosis was made during skin cancer screening, is essential.

## Conclusion

Although proportional changes in stage distribution were found in our analyses, there were no distinct proportional changes which can be directly attributed to the introduction of skin cancer screening in 2008. No direct effect of skin cancer screening up to the year 2011 could therefore be shown.

## Additional files

Additional file 1: Figure S1. Flow chart of inclusion and exclusion of malignant melanoma cases diagnosed between 2002 and 2011 in Germany (TIF 135 kb)
Additional file 2: Table S1. pT stage by UICC Stage of malignant melanoma patients diagnosed between 2002 and 2011 (*N* = 61 895) (DOCX 38 kb)
Additional file 3: Table S2. Malignant melanoma patients aged 35 years and above by age at diagnosis, sex, UICC stage, year of diagnosis, place of residence and ‘diagnosis during screening’, *N* = 34 739 (UICC 0 and X excluded) (DOCX 40 kb)
Additional file 4: Table S3. Relative 5-year survival of malignant melanoma patients diagnosed between 2002 and 2011, overall (UICC 0-IV, X) (*N* = 60 672) and for patients with invasive tumours (UICC I – IV, X) stratified by age, sex, UICC stage, ‘diagnosis during screening’ and place of residence (*N* = 49 351) (DOCX 39 kb)

## Abbreviations

ADT: Working group of German tumour centres and clinical cancer registries
APC: Annual percent change
CI: Confidence interval
DCO: Death certificate only
GEKID: Association of population-based cancer registries in Germany
ICD-O: International Classification of Diseases for Oncology (3rd Edition)
ICD-10: International Statistical Classification of Diseases and Related Health Problems (10th revision)
OR: Odds ratio
ref: Reference category
TNM: Tumour-Node-Metastasis classification of malignant tumours
UICC: Union internationale contre le cancer
