# Supplementary figures and images for: Tumour stage distribution and survival of malignant melanoma in Germany 2002–2011
Source: BMC Cancer. 2016 Dec 5;16:936. doi: 10.1186/s12885-016-2963-0 (PMC5139127; doi:10.1186/s12885-016-2963-0)

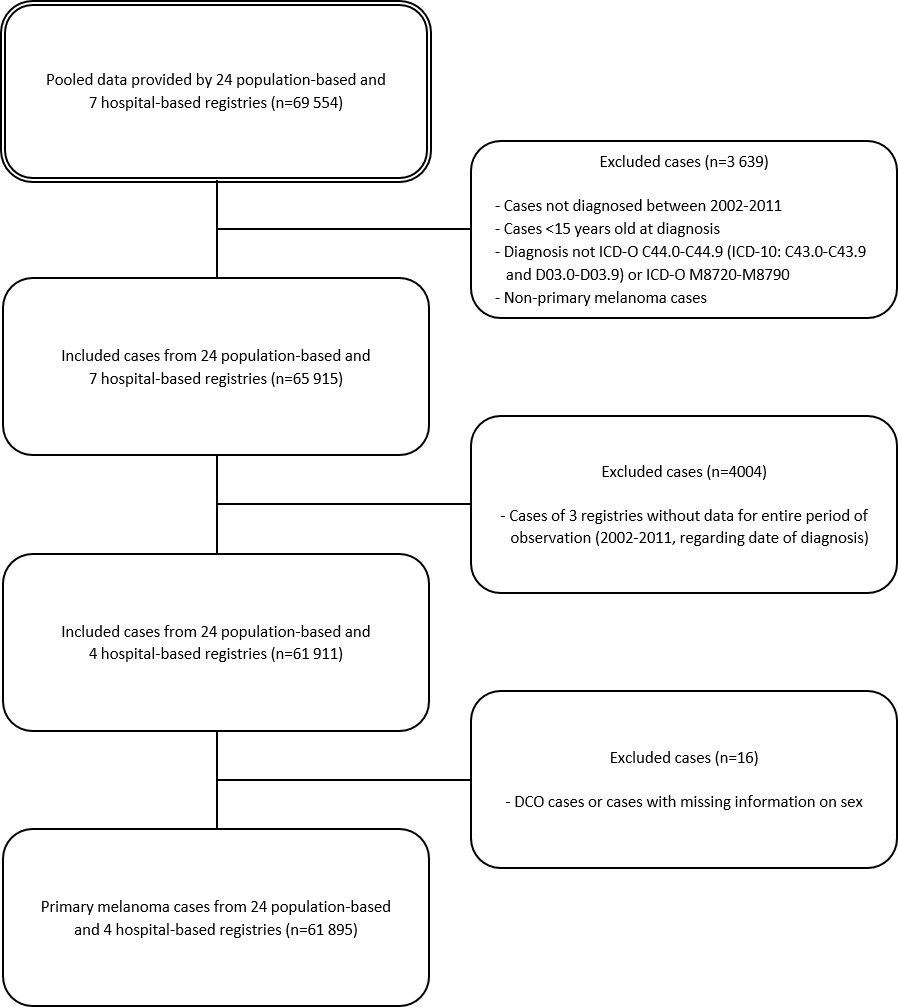

Supplement: Additional file 1: Figure S1. — Flow chart of inclusion and exclusion of malignant melanoma cases diagnosed between 2002 and 2011 in Germany (TIF 135 kb) [file 12885_2016_2963_MOESM1_ESM.tif]
