# Supplementary material for: Tumour stage distribution and survival of malignant melanoma in Germany 2002–2011
Source: BMC Cancer. 2016 Dec 5;16:936. doi: 10.1186/s12885-016-2963-0 (PMC5139127; doi:10.1186/s12885-016-2963-0)
Supplement: Additional file 2: Table S1. — pT stage by UICC Stage of malignant melanoma patients diagnosed between 2002 and 2011 (N = 61 895) (DOCX 38 kb) [file 12885_2016_2963_MOESM2_ESM.docx]

**Supplement Table 2: pT stage by UICC Stage of malignant melanoma patients diagnosed between 2002 and 2011 (N=61 895)**

|  |  | **Stage** | | | | | | **Total** |
| --- | --- | --- | --- | --- | --- | --- | --- | --- |
|  |  | **UICC 0** | **UICC I** | **UICC II** | **UICC III** | **UICC IV** | **UICC X** | **N (%)*** |
| **Stratum** | | **N (%)*** | | | | | |  |
| Total |  | 11 449 (100.0) | 25 681 (100.0) | 7 352 (100.0) | 3 555 (100.0) | 1 359 (100.0) | 12 499 (100.ß) | 61 895 (100.0) |
| pT stage | in situ | 11 449 (100.0) | 0 (0.0) | 0 (0.0) | 0 (0.0) | 0 (0.0) | 0 (0.0) | 11 449 (18.5) |
|  | 1 | 0 (0.0) | 19 937 (77.6) | 0 (0.0) | 248 (7.0) | 93 (6.8) | 4 502 (36.0) | 24 780 (40.0) |
|  | 2 | 0 (0.0) | 5 659 (22.0) | 880 (12.0) | 763 (21.5) | 91 (6.7) | 834 (6.7) | 8 227 (13.3) |
|  | 3 | 0 (0.0) | 0 (0.0) | 4 269 (58.1) | 1 145 (32.2) | 177 (13.0) | 794 (6.4) | 6 385 (10.3) |
|  | 4 | 0 (0.0) | 0 (0.0) | 2 186 (29.7) | 1 199 (33.7) | 419 (30.8) | 693 (5.5) | 4 497 (7.3) |
|  | X | 0 (0.0) | 85 (0.3) | 17 (0.2) | 200 (5.6) | 579 (42.6) | 5 676 (45.4) | 6 557 (10.6) |

*percentages refer to column
