# Supplementary material for: Tumour stage distribution and survival of malignant melanoma in Germany 2002–2011
Source: BMC Cancer. 2016 Dec 5;16:936. doi: 10.1186/s12885-016-2963-0 (PMC5139127; doi:10.1186/s12885-016-2963-0)
Supplement: Additional file 3: Table S2. — Malignant melanoma patients aged 35 years and above by age at diagnosis, sex, UICC stage, year of diagnosis, place of residence and ‘diagnosis during screening’, N = 34 739 (UICC 0 and X excluded) (DOCX 40 kb) [file 12885_2016_2963_MOESM3_ESM.docx]

**Supplement Table 3: Malignant melanoma patients aged 35 years and above by age at diagnosis, sex, UICC stage, year of diagnosis, place of residence and diagnosis during screening, N=34 739 (UICC 0 and X excluded)**

|  |  | **Diagnosis during Screening** | | | **Total** |
| --- | --- | --- | --- | --- | --- |
| **Stratum** |  | **Yes** | **No** | **Unknown** | **N (%)**** |
|  |  | **N (%)*** | | |  |
| Total |  | 586 (1.7) | 10 925 (31.4) | 23 228 (66.9) | 34 739 (100.0) |
| Age at diagnosis (years) | 35-49 | 134 (1.7) | 1 997 (25.7) | 5 626 (72.5) | 7 757 (22.3) |
|  | 50-64 | 207 (2.0) | 3 295 (31.3) | 7 033 (66.8) | 10 535 (30.3) |
|  | 65-79 | 207 (1.6) | 4 409 (33.9) | 8 407 (64.6) | 13 023 (37.5) |
|  | ≥80 | 38 (1.1) | 1 224 (35.7) | 2 162 (63.1) | 3 424 (9.9) |
| Sex | Male | 316 (1.7) | 5 660 (31.2) | 12 160 (67.0) | 18 136 (52.2) |
|  | Female | 270 (1.6) | 5 265 (31.7) | 11 068 (66.7) | 16 603 (47.8) |
| UICC stage | I | 519 (2.2) | 7 154 (30.9) | 15 446 (66.8) | 23 119 (66.6) |
|  | II | 49 (0.7) | 2 481 (35.4) | 4 476 (63.9) | 7 006 (20.2) |
|  | III | 16 (0.5) | 918 (27.8) | 2 369 (71.7) | 3 303 (9.5) |
|  | IV | 2 (0.2) | 372 (28.4) | 937 (71.5) | 1 311 (3.8) |
| Year of diagnosis | 2002 | 19 (0.7) | 908 (32.6) | 1 860 (66.7) | 2 787 (8.0) |
|  | 2003 | 17 (0.6) | 935 (32.5) | 1 921 (66.9) | 2 873 (8.3) |
|  | 2004 | 20 (0.6) | 922 (29.8) | 2 150 (69.5) | 3 092 (8.9) |
|  | 2005 | 18 (0.5) | 918 (28.0) | 2 340 (71.4) | 3 276 (9.4) |
|  | 2006 | 32 (1.0) | 1 011 (30.6) | 2 263 (68.5) | 3 306 (9.5) |
|  | 2007 | 26 (0.7) | 1 052 (29.9) | 2 436 (69.3) | 3 514 (10.1) |
|  | 2008 | 71 (1.7) | 1 280 (31.4) | 2 722 (66.8) | 4 073 (11.7) |
|  | 2009 | 149 (3.9) | 1 264 (32.9) | 2 432 (63.3) | 3 845 (11.1) |
|  | 2010 | 108 (2.7) | 1 312 (32.5) | 2 618 (64.8) | 4 038 (11.6) |
|  | 2011 | 126 (3.2) | 1 323 (33.6) | 2 486 (63.2) | 3 935 (11.3) |
| Place of residence | Eastern Germany | 326 (2.3) | 8 906 (63.8) | 4 717 (33.8) | 13 949 (40.2) |
|  | Western Germany | 260 (1.3) | 2 019 (9.7) | 18 511 (89.0) | 20 790 (59.8) |

*percentages refer to row, **percentages refer to column
