# Supplementary material for: Tumour stage distribution and survival of malignant melanoma in Germany 2002–2011
Source: BMC Cancer. 2016 Dec 5;16:936. doi: 10.1186/s12885-016-2963-0 (PMC5139127; doi:10.1186/s12885-016-2963-0)
Supplement: Additional file 4: Table S3. — Relative 5-year survival of malignant melanoma patients diagnosed between 2002 and 2011, overall (UICC 0-IV, X) (N = 60 672) and for patients with invasive tumours (UICC I – IV, X) stratified by age, sex, UICC stage, ‘diagnosis during screening’ and place of residence (N = 49 351) (DOCX 39 kb) [file 12885_2016_2963_MOESM4_ESM.docx]

**Supplement Table 1: Relative 5-year survival of malignant melanoma patients diagnosed between 2002 and 2011, overall (UICC 0-IV, X) (N=60 672) and for patients with invasive tumours (UICC I – IV, X) stratified by age, sex, UICC stage, diagnosis during screening and place of residence (N=49 351)**

| **Stratum** | | **N** | **Relative 5-year survival in %** | | **Log- Rank test (p value)** |
| --- | --- | --- | --- | --- | --- |
|  |  |  | **Survival rate** | **95% CI**** |  |
| Overall |  | 60 672 | 85.8 | [85.3; 86.2] | - |
| *In situ* tumour (UICC 0) | Yes | 11 321 | 97.5 | [96.6; 98.5] | <0.0001 |
|  | No | 49 351 | 83.4 | [82.8; 83.9] | reference |
| Age at diagnosis (years)* | 15-34 | 4 064 | 94.6 | [93.7; 95.6] | <0.0001 |
|  | 35-49 | 9 871 | 89.6 | [88.8; 90.5] | <0.0001 |
|  | 50-64 | 13 509 | 85.2 | [84.3; 86.0] | <0.0001 |
|  | 65-79 | 17 056 | 78.4 | [77.4; 79.4] | reference |
|  | ≥80 | 4 851 | 66.9 | [64.8; 69.1] | <0.0001 |
| Sex* | Male | 24 741 | 80.5 | [79.6; 81.4] | reference |
|  | Female | 24 610 | 86.2 | [85.5; 86.8] | <0.0001 |
| UICC stage* | I | 25 446 | 96.8 | [96.2; 97.5] | reference |
|  | II | 7 256 | 74.2 | [72.8; 75.6] | <0.0001 |
|  | III | 3 479 | 56.7 | [54.6; 58.8] | <0.0001 |
|  | IV | 1 331 | 18.4 | [15.8; 21.1] | <0.0001 |
|  | X | 11 839 | 76.6 | [75.2; 77.9] | <0.0001 |
| Diagnosis during screening* | Yes | 745 | 94.2 | [89.9; 98.5] | <0.0001 |
|  | No | 14 026 | 84.1 | [83.0; 85.1] | reference |
|  | Unknown | 34 580 | 82.9 | [82.3; 83.6] | 0.6813 |
| Place of residence* | Eastern Germany | 18 720 | 83.9 | [83.0; 84.8] | reference |
|  | Western Germany | 30 631 | 83.0 | [82.4; 83.7] | 0.3057 |

* only patients with invasive tumours (UICC I-IV, X), ** CI=Confidence interval
